# 3Omics: a web-based systems biology tool for analysis, integration and visualization of human transcriptomic, proteomic and metabolomic data

**DOI:** 10.1186/1752-0509-7-64

**Published:** 2013-07-23

**Authors:** Tien-Chueh Kuo, Tze-Feng Tian, Yufeng Jane Tseng

**Affiliations:** 1 Graduate Institute of Biomedical Electronics and Bioinformatics, National Taiwan University, Taipei, Taiwan; 2 The Metabolomics Core Laboratory, Center of Genomic Medicine, , Taipei, Taiwan; 3 Department of Computer Science and Information Engineering, National Taiwan University, Taipei, Taiwan

**Keywords:** Visualization, Omics integration, Systems biology, Transcriptomics, Proteomics, Metabolomics, Analysis

## Abstract

**Background:**

Integrative and comparative analyses of multiple transcriptomics, proteomics and metabolomics datasets require an intensive knowledge of tools and background concepts. Thus, it is challenging for users to perform such analyses, highlighting the need for a single tool for such purposes. The 3Omics one-click web tool was developed to visualize and rapidly integrate multiple human inter- or intra-transcriptomic, proteomic, and metabolomic data by combining five commonly used analyses: correlation networking, coexpression, phenotyping, pathway enrichment, and GO (Gene Ontology) enrichment.

**Results:**

3Omics generates inter-omic correlation networks to visualize relationships in data with respect to time or experimental conditions for all transcripts, proteins and metabolites. If only two of three omics datasets are input, then 3Omics supplements the missing transcript, protein or metabolite information related to the input data by text-mining the PubMed database. 3Omics’ coexpression analysis assists in revealing functions shared among different omics datasets. 3Omics’ phenotype analysis integrates Online Mendelian Inheritance in Man with available transcript or protein data. Pathway enrichment analysis on metabolomics data by 3Omics reveals enriched pathways in the KEGG/HumanCyc database. 3Omics performs statistical Gene Ontology-based functional enrichment analyses to display significantly overrepresented GO terms in transcriptomic experiments. Although the principal application of 3Omics is the integration of multiple omics datasets, it is also capable of analyzing individual omics datasets. The information obtained from the analyses of 3Omics in Case Studies 1 and 2 are also in accordance with comprehensive findings in the literature.

**Conclusions:**

3Omics incorporates the advantages and functionality of existing software into a single platform, thereby simplifying data analysis and enabling the user to perform a one-click integrated analysis. Visualization and analysis results are downloadable for further user customization and analysis. The 3Omics software can be freely accessed at http://3omics.cmdm.tw.

## Background

The development and integration of transcriptomics, proteomics and metabolomics comprise the majority of current systems biology studies and provide a significant capacity for the investigation of biological mechanisms and their associations with diseases in a “big picture” approach [1-3]. Integrated analyses of transcriptomics and metabolomics [4-6] have revealed significant associations between gene and metabolite expression profiles. Nam et al. [7] demonstrated the increased coherence and robustness of newly discovered breast cancer biomarkers by utilizing both gene expression profiles and metabolic profiles to validate biomarker significance. Su et al. [8] combined metabolomic and transcriptomic analyses of the NCI-60 dataset to identify significant tissue/organ-specific metabolome and transcriptome features that are related to various cancer types. Using integrated metabolomic and transcriptomic analysis, Su et al. [8] identified biologically meaningful gene-metabolite associations, including several abnormal gene-metabolite relationships that were directly linked to known gene mutations and copy-number variations in the corresponding cell lines. Improved data visualization tools are required to efficiently incorporate these vast amounts of data into an intuitive, integrated and knowledge-based environment.

### Current tools for integrating omics data

Current visualization tools for integrating omics data can be loosely classified as biological network-based or pathway-based tools [9]. Biological networks reveal hidden patterns in the original unstructured data by transforming raw data into logically structured and visually tangible representations—with nodes representing genes, proteins and metabolites and with edges indicating interactions between nodes or clusters that share similar molecular functions. The majority of network-based visualization tools, e.g., VANTED [10], VisAnt [11], and Metscape2 [12], are integrated with public databases. Arena3D further allows users to visualize biological networks in three dimensions [13]. For small networks, interactive editing is often performed manually. For large networks, however, it is easier to utilize automated layout tools, such as Cytoscape [14], NAViGaTOR [15], and Cerebral [16].

Alternatively, pathway visualization tools allow users to explore the biochemical activities found in experimental datasets along different interactive pathways. Pathguide [17] provides an overview of over 190 web-accessible biological pathway and network databases. Arakawa et al. [18] developed a KEGG-based pathway visualization tool for KEGG pathway databases [19]. Pathway-level visualization of different omics data representations allows users to capture systematic properties of biochemical activities. Paintomics [20] focuses on gene expression and metabolite concentration data and displays the data on KEGG pathway maps. ProMeTra [21] accepts annotated images in SVG format and is capable of displaying dynamic data. KaPPa-View [22] and MapMan [23] display metabolite and transcript levels for predefined pathway blocks in plants. ChromeTracks for MAYDAY [24] allows for the visualization of expression data with any metadata within a genomic context. PaVESy [25] builds customized pathways from user-provided proteins and metabolites.

### Comparison of 3Omics with other software

A summary and comparison of accessibility, data export and exchange, functions and utilities, and source data types found in the currently available software with those in 3Omics is presented in Table 1.

**Table 1:** Features of integrated analysis tools according to requirements identified in 3Omics

**Tool name**	**Ref**	**R1**	**R1**	**R1**	**R2**	**R2**	**R3**	**R3**	**R3**	**R3**	**R3**	**R3**	**R3**	**R3**	**R3**	**R4**	**R4**	**R4**	**R4**
		**Installation-free**	**Free for academics**	**No registration**	**Save as SVG/SIF**	**Processed data downloaded**	**Human-specific analysis**	**Network visualization**	**Omics data integrated analysis**	**Correlation analysis**	**Coexpression profiling**	**Phenotype mapping**	**Pathway enrichment analysis**	**GO enrichment analysis**	**Links to external DBs**	**Multiple conditions and time-series data**	**Transcriptome data**	**Proteome data**	**Metabolome data**
3Omics		●	●	●	●	●	●	●	●	●	●	●	●	●	●	●	●	●	●
VANTED	[10]		●	●		●	○	●								●	●	●	●
VisANT	[11]	○	●	○	●	●	○	●						○	●		●	○	○
NAViGaTOR	[15]		●	●			○	●									○	○	○
Cerebral	[16]		●	●	●		○	●									○	○	○
Paintomics	[20]	●	●	●	●	●	○		○			○			●		●		●
ProMeTra	[21]	●	●	●	●	●	○		○			○			●	●	●	●	●
KappaView	[22]	●	●	●	●	●			●			○			●	●	●		●
MapMan	[23]		●	●															
ChromeTracks	[24]		●	●	●	●	○		○		○				●	●	●		●
PaVESy	[25]	●	●	●		●	○					○			●		○	○	○
IPA	[26]				●		●	●	●	●	●	●	●	●	●	●	●	●	●

(R1) Accessibility, (R2) Data Export and Exchange, (R3) Functions and Utilities, (R4) Data Source Type. Filled circles indicate tools with the corresponding features. Empty circles indicate tools with partial functionality.

3Omics is specifically designed for the analysis of human data because all of the incorporated transcripts, proteins, metabolites, pathways, and gene information are human-specific. The majority of the existing software described in Table 1 is designed for a wide variety of specific organisms, except for KaPPa-View and MapMan, which are plant-specific tools. Ingenuity Pathway Analysis (IPA) [26] provides the full suite of omics analysis tools; however, users may not have access to this commercial software. While IPA supports various network visualization and analyses, 3Omics simplifies the data analysis by combining the advantages and operations of several existing systems and packages into a single platform. 3Omics accepts multiple experimental conditions or time-dependent transcriptomics data, proteomics data or metabolomics data. Users can perform correlation analysis, coexpression profiling, phenotype mapping, pathway enrichment analysis and GO enrichment analysis on each dataset via a single platform. These features enable users to perform an integrated analysis with one click, a versatile function not featured in other software.

## Implementation

### System overview

3Omics is a platform-independent web application constructed with Perl and PHP scripts and running on a Linux-based Apache web server. A typical session workflow is illustrated in Additional 1: Figure S1. When users upload experimental data via the 3Omics web interface, the server immediately computes correlation coefficients, coexpression values and pathway enrichment scores. Related information from publicly accessible databases, such as iHOP (information hyperlinked over proteins) [27,28], KEGG [19], HumanCyc [29], DAVID [30], Entrez Gene [31], OMIM and UniProt [32], are automatically incorporated and stored in an internal database. To maintain up-to-date information, the internal database is updated with new data each month (from KEGG, HumanCyc, Entrez Gene, OMIM, and UniProt) or queried immediately (iHOP and DAVID).

Users can export network images in SVG or SIF formats. SVG is an XML-based file format for describing two-dimensional vector graphics that is compatible with multiple platforms. Processed SIF files can be imported and edited in Cytoscape. 3Omics users can also download all of the processed data for further analysis. All analyzed data and network/pathway images can be downloaded in PNG or SVG format. Detailed descriptions of the supported data formats are found on the 3Omics online help page. All uploaded data files are temporarily stored during a 3Omics session and automatically deleted after processing to safeguard data confidentiality. In the following subsections, we briefly explain the methods used in 3Omics.

### Summary of 3Omics features

**Figure 1:**
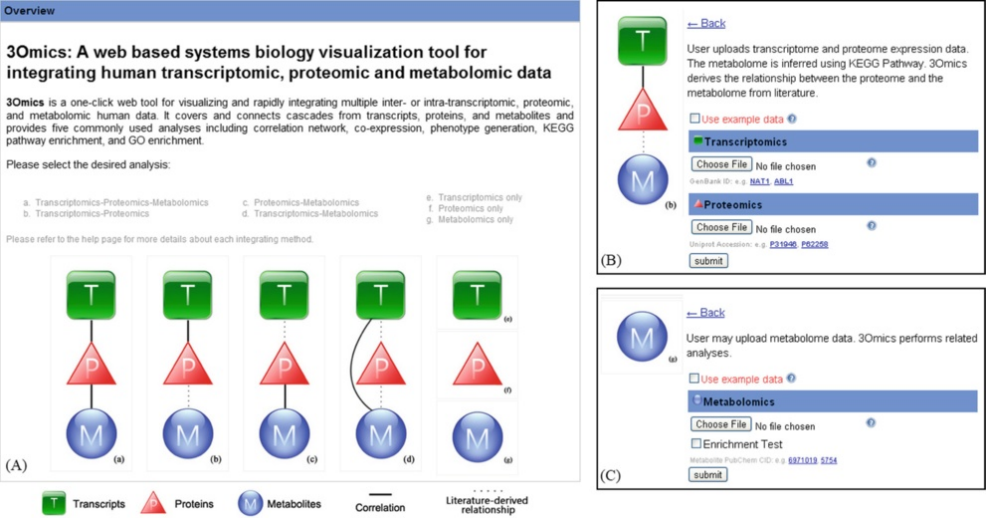
**3Omics User interface. (A)** 3Omics implements seven inter-omic analyses: **(a)** Transcriptomics-Proteomics-Metabolomics, **(b)** Transcriptomics-Proteomics, **(c)** Proteomics-Metabolomics, **(d)** Treanscriptomics-Metabolomics, and intra-omics analyses, such as **(e)** Transcriptomics, **(f)** Proteomics, and **(g)** Metabolomics. Users select the desired analysis by selecting the corresponding icon. **(B)** Interface for the Transcriptomics-Proteomics analysis. **(C)** Interface for the Metabolomics analysis.

3Omics offers four types of multiple omics analysis depending on the data provided by the user (see options a, b, c and d on the 3Omics homepage in Figure 1): Transcriptomics – Proteomics - Metabolomics (T – P - M) analysis, Transcriptomics - Proteomics (T - P) analysis, Proteomics - Metabolomics (P - M) analysis, and Transcriptomics - Metabolomics (T - M) analysis. 3Omics also offers analysis in single-omics mode to reveal “intra-omics” relationships (see options e, f, and g in Figure 1).

3Omics analysis requires the use of transcript, protein, or metabolite IDs and their corresponding variations (e.g., concentration or intensity levels) under specific experimental conditions (e.g., different times, nucleic magnetic resonance shifts (in parts per million) or mass spectrometry mass-to-charge ratios). Acceptable IDs include Entrez Gene IDs, UniprotKB IDs and PubChem CIDs [33]. Users can also utilize the 3Omics Name-ID Converter to match gene, protein and metabolite names with their corresponding IDs. Once users select an analysis method, a data input page is dynamically generated to upload the required data. For example, when a user would like to perform a T-P analysis, 3Omics loads the page shown in the upper-right corner of Figure 1. T and P data from large-scale biochemical experiments are then uploaded in a comma-separated value format. Where different data integrations may require different analyses, 3Omics, in general terms, provides correlation, coexpression, phenotype, pathway enrichment, and GO enrichment analyses.

**Figure 2:**
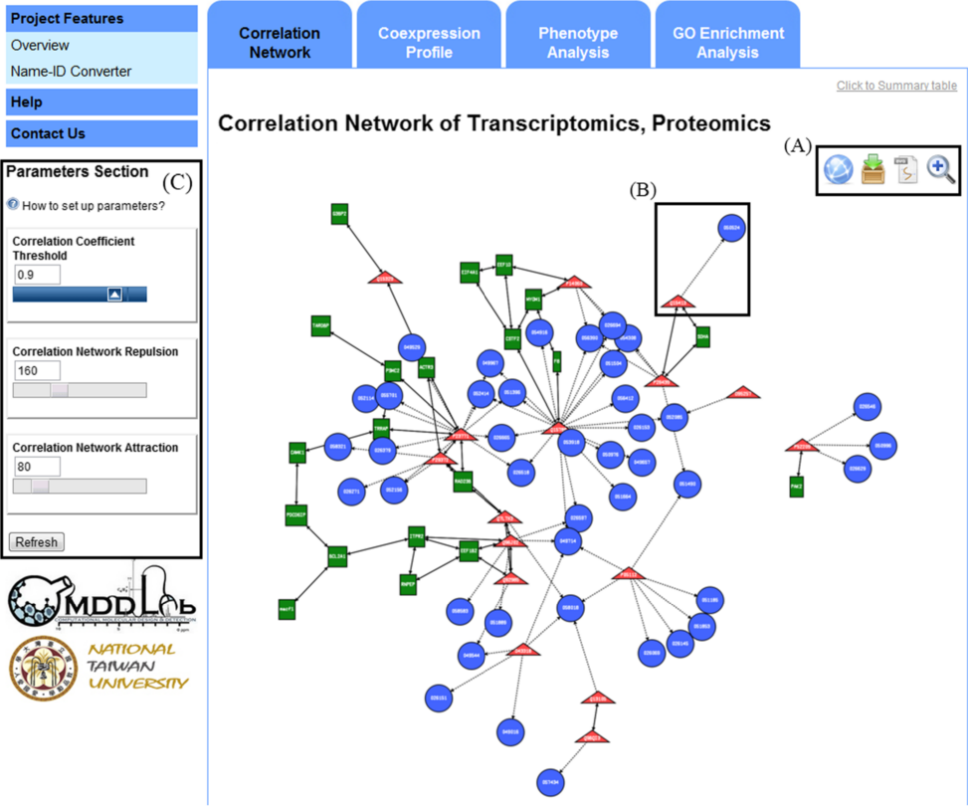
**3Omics-generated Correlation network analysis.** Features include the following: **(A)** toggling zoom/explore mode, saving as SVG format, downloading the full-size image and SIF files for Cytoscape import; **(B)** literature-derived edges are presented as dotted lines; **(C)** adjusting parameters to customize the correlation network.

Table 2 lists the various data integration methods and analyses incorporated into 3Omics. When a user possesses transcriptomics, proteomics and metabolomics data, all analyses are performed. When only two of the three omics datasets are available, 3Omics supplements missing transcript, protein and metabolite information related to the user-input data by text-mining biomedical literature from iHOP to generate literature-derived objects and relationships for correlation analysis (see the dotted line depicting the literature-derived relationship in Figure 2B). Coexpression analysis is available for all omics data types. For transcriptomic or proteomic datasets, phenotype- and GO-based enrichment analyses can be performed. In addition, pathway enrichment analysis can be employed to map metabolite data to a KEGG/HumanCyc pathway to determine significant or differentially expressed metabolites that may play vital roles in the corresponding biological pathway.

**Table 2:** Types of analyses available in 3Omics

**Type of analysis**	**Correlation analysis**	**Coexpression analysis**	**Phenotype analysis**	**Pathway enrichment analysis**	**Gene ontology enrichment analysis**
**T-P-M**	Yes	Yes	Yes	Yes	Yes
**T-P**	Yes, M is derived from the literature.	Yes	Yes	No	Yes
**P-M**	Yes, T is derived from the literature.	Yes	Yes	Yes	No
**T-M**	Yes, P is derived from the literature.	Yes	Yes	Yes	Yes
**T**	Yes	Yes	Yes	No	Yes
**P**	Yes	Yes	Yes	No	No
**M**	Yes	Yes	No	Yes	No

T: Transcriptomics dataset, P: Proteomics dataset, and M: Metabolomics dataset. T, P, or M linked with hyphens stands for two or three omics dataset are provided by users.

Analyses provided in 3Omics when users input different omics dataset. For example, correlation analysis, coexpression analysis, phenotype analysis, and GO enrichment analysis are provided for users input transcriptomics-proteomics datasets.

### Correlation analysis

3Omics incorporates the “corr” function from R [34] to compute the Pearson correlation coefficient (PCC). PCC is widely used and accepted as a measure of correlation in systems biology. Nodes and edges are stored in a Graph Description Language (GDL) format and sent to the network generator, aiSee3 (AbsInt, Angewandte Informatik GmbH, Saarbrücken, Germany). A force-directed layout algorithm is utilized to generate visualizations. The correlation coefficient threshold and the repulsion and attraction parameters can be adjusted for better visualization and are set by default to 0.9, 160 and 80, respectively (see the lower-left corner of Figure 2).

To generate a correlation network, the PCCs are calculated from two sets of expression values for two entities (inter- or intra-omics data). The PCC correlation matrix is calculated for the omics data, which are then used for visualization. Correlation networks can be generated automatically, and substances can be clustered according to similar behavior over time or into different experimental groups. Nodes denoted by squares, triangles and circles represent transcripts, proteins and metabolites, respectively. Transcript, enzyme and metabolite can be presented in the context of the correlation network. Correlated relationships (PCC > 0.9) are represented by solid lines, and the text-mining results between pairs of input molecules and literature-derived molecules are indicated by dotted lines. The results can be downloaded from the web interface. Navigation functions support visual exploration of the data-enriched networks (Figure 2A).

#### Literature-derived relationships in the correlation analysis

When only two of the three omics datasets are available for correlation network analysis, 3Omics supplies the missing omics information using the following approach. First, 3Omics identifies related transcript-protein, protein-metabolite, or transcript-metabolite pairs by incorporating 48,631 human genes from NCBI Entrez Gene, 20,370 human proteins from UniProt, and 16,339 metabolite entries from KEGG Compound [19] into an internal, relational database. Original data from the May 8, 2012 snapshot were downloaded using NCBI EFetch and UniProt as well as the KEGG FTP site. Each entity in the MySQL database should contain a transcript-protein-metabolite relationship. When an entity contains only transcript-protein, protein-metabolite, or transcript-metabolite pairs, 3Omics can rapidly identify the missing transcripts, proteins or metabolites and their potential relationships for correlation analysis.

Once the missing information is identified, 3Omics uses the transcripts or proteins to search iHOP. The missing omics data are supplied from the iHOP results. For example, transcriptomics and proteomics data are inputs, and 3Omics searches for protein-metabolite pairs. The missing metabolomics data are recovered by text mining of the iHOP results. Relationships described in the literature are depicted as dotted edges in the plot, as shown in Figure 2.

### Coexpression analysis

Coexpression analysis is performed using the R statistical programming language [34]. Heatmaps are generated using the R language gplots package [35]. Rows display the expression of input molecules, and columns display the expression differences between experimental groups, such as treatment/control groups or time-series experiments. Each cell in the resulting image is “heat colorized” based on the input expression value. Cyan indicates the lowest expression value, and pink indicates the highest expression value. Row edges are color coded to indicate their omics data source types. Heatmap dendrograms are added to the top and left side of the heatmaps to display similarities among rows or columns. Dendrograms on the top and left side of the image display the similarities of the input molecules (each row represents a transcript, protein, or metabolite) or experimental groups (each column represents a treatment group or control group). Dissimilarity coefficients between rows and columns are computed as the Euclidean distance, where the closest rows/columns connected by dendrograms have the most similar expression profiles.

### Phenotype analysis

A phenotype is defined as any observable characteristic or trait of an organism arising from gene expression, the influence of environmental factors, and interactions between gene expression and environmental factors. A total of 21,746 phenotypes listed in OMIM from the March 27, 2012 snapshot were downloaded from the OMIM website and stored in the internal 3Omics database. The OMIM data are used to identify genes and genetic disorders based on information that relates genes in the human genome with specific phenotypes.

Phenotype analysis is not available for metabolomics datasets alone because no transcriptomic or proteomics data are available.

### Pathway enrichment analysis

A total of 499 human pathways from KEGG Pathway and 793 human pathways from HumanCyc were downloaded and stored in the internal 3Omics database (Release 62.0 and Version 16.0). HumanCyc provides more than 250 human pathways with literature-based curation for at least one year by experts. The pathways in HumanCyc are small and similar to biologically functional units. Therefore, enriched pathways from HumanCyc provide meaningful information from input metabolomics data. Two modes are available in 3Omics’ KEGG pathway enrichment analysis: normal and enrichment. The normal mode displays user-provided metabolites via simple metabolite mapping to a pathway from the KEGG Pathway database. The enrichment mode requires users to upload two datasets: (A) a metabolite set and (B) a significantly changed metabolite set. Significantly enriched pathways are identified with a hypergeometric test for a given list of metabolites. For example, there are *N* metabolites in set A and *n* metabolites in set B, and there are *m* metabolites in set A and *i* metabolites in set B in a given KEGG human pathway. The probability of the occurrence of *x* or fewer metabolites within set B in a given pathway is calculated by hypergeometric distribution [36] according to the following formula:

$$Ρ=\sum_{i-x}^{n}\frac{\left(\underset{i}{m}\right)\left(\underset{n-i}{N-m}\right)}{\left(\underset{n}{N}\right)}$$

The hypergeometric test is a standard method for calculating pathway enrichment. Note that when a large population (*N*) is selected and the total number of mapped metabolites in set A (*m*) is also large, the cumulative probability in the hypergeometric test will be very high.

The pathway enrichment analysis is available for proteomics-metabolomics, transcriptomics-metabolomics, transcriptomics-proteomics-metabolomics, and single metabolomics analyses.

### Gene ontology-based enrichment analysis

GO-based functional enrichment analysis is performed through the DAVID knowledgebase Application Platform Interface (API). Three independent GOs are included: (i) biological processes, (ii) cellular components, and (iii) molecular functions. The input transcripts are used in 3Omics to calculate the p-value and FDR (False Discovery Rate) of each GO term using a modified Fisher’s exact test in the DAVID API. The enriched GO terms associated with the given Entrez Gene IDs are reported in 3Omics. By default, enriched terms with p-values less than 0.05 are presented in an interactive bar chart generated with Google Chart Tools [37].

Using GO enrichment analysis, only the enriched terms are displayed, thus avoiding the display of general terms, such as “cellular component” or “metabolic process”, which are of limited use because many transcripts and proteins can be mapped to them. GO-based enrichment analysis requires transcriptomics data to calculate the GO-term enrichment; therefore, GO-based functional enrichment analysis is available for transcriptomics-proteomics, transcriptomics-metabolomics, transcriptomics-proteomics-metabolomics and single transcriptomics analyses. GO enrichment analysis allows users to explore genes represented by GO terms with automated organization functionality, thereby avoiding the need for manual editing.

## Results and discussion

### Case studies

Two cases were selected to demonstrate the main functions of 3Omics and its usefulness for omics analysis: a transcriptome and proteome dataset for an acute promyelocytic leukemia human cell line [38] and a urinary metabolome dataset from MetPA [39].

#### Case study 1: Integrated transcriptome and proteome analysis of retinoic acid/arsenic trioxide-induced cell differentiation/apoptosis in promyelocytic leukemia

To demonstrate transcriptome-proteome analysis with literature-derived metabolites, transcriptome and proteome data from promyelocytic leukemia cells treated with retinoic acid (RA), arsenic trioxide (ATO), or a combination of the two were selected for this case study. The experiments were performed with the NB4 cell line, a human acute promyelocytic leukemia (APL) cell line. In this case study, correlation network and GO-enrichment analyses were used to demonstrate the user interface and functionality.

A correlation network can be constructed by calculating the PCCs between pairs of entities to uncover possible interactions in omics data. Figure 2 presents the correlation network that was automatically generated by 3Omics for the integrated proteome and transcriptome, showing highly correlated transcript-transcript, transcript-protein and protein-protein relationships (PCCs > 0.9). In this visualization of the correlation network and clusters of highly correlated molecules (for example, transcripts (green rectangles), proteins (red triangles), or literature-derived metabolites (blue circles) in Case Study 1), the transcripts and proteins sharing similar expression profiles in each treatment and time point are readily observed. The clusters in the correlation network are also shown in a summary table (Additional 2: Figure S2). Clusters are ranked by descending size (the number of nodes in a cluster). The largest cluster has a size of nineteen input molecules, including BCL2A1, PDCD6IP, TARDBP, TRRAP, and RAD23B, which are highly correlated. In the second cluster, seven molecules, including EIF4A1, EEF1D, and MYOM1, share similar PCCs. This cluster implies that these molecules may be expressed under the same regulation mechanism or related ones. The correlations of input molecules analyzed by 3Omics are consistent with those described in the original report [38]. The transcriptomic expression levels of BCL2A1, PDCD6IP, and TARDBP are up-regulated, as are the proteomic expression levels of TRRAP and RAD23B. These genes and proteins are highly correlated, suggesting that the results of the correlation network analysis provide reliable information with a one-click analysis. PCCs also display the significance as a measure of the correlation between input molecules in 3Omics. The functionality of the molecules is then revealed in text-mining results and the GO enrichment analysis, and the phenotype analysis is used for human phenotypic mapping.

Blue circles in the correlation network indicate metabolites, and dotted edges between two nodes indicate relationships described in the literature. The literature-derived metabolite relationships provide insight into the possible mechanism or phenotype. One input protein may be associated with many literature-derived metabolites. One literature-derived metabolite may likewise be associated with many input proteins. Thus, both proteins and literature-derived metabolites may appear several times in the association table (Additional 3: Figure S3). To further understand the relationship between the input proteins and literature-derived metabolites, the user can click on the input gene/protein ID (For example, click ‘P14060’ in the first column in Additional 3: Figure S3) to search external link in iHOP for further information. In iHOP, relationships among proteins and metabolites are then reported as sentences and both protein-metabolite and protein-disease relationships can be found in iHOP. For example, this case study search revealed that 5-alpha-dihydrotestosterone activates androgen receptors and influences steroid receptor coactivator-1 (SRC-1 or NCOA1, UniProt protein ID: Q15788) in promyelocytic leukemia, as noted on the NCOA1 page in iHOP [40]. In addition, the association of breast/prostate cancer and NCOA1 is found in iHOP [41,42]. With literature-derived results, the relationships among input molecules and among molecules and diseases are easily found. However, such relationships often require further experimental confirmation, similar to all text-mining-based discoveries.

**Figure 3:**
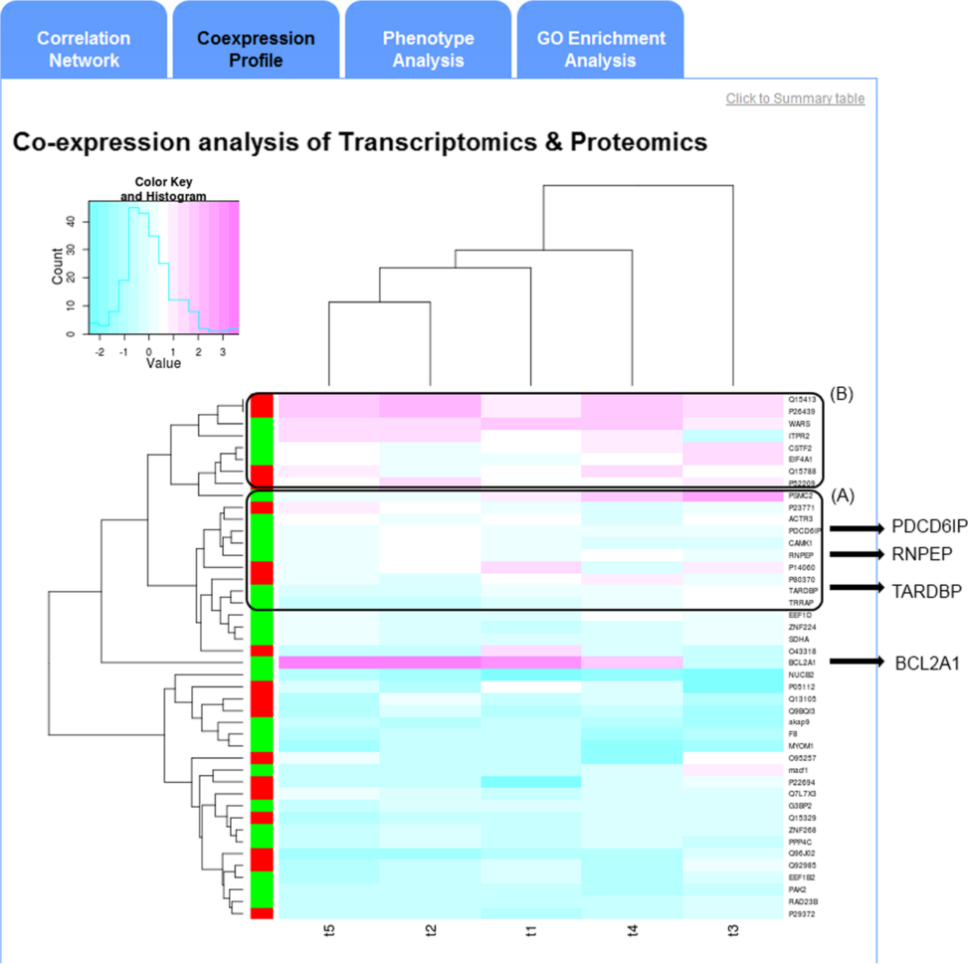
**3Omics-generated coexpression profile.** An example coexpression profile for a Transcriptomics-Metabolomics analysis is shown. **(A)** Molecules in the largest cluster of the correlation analysis have highly similar expression profiles. **(B)** Molecules in the second-largest cluster also have highly similar expression profiles. Pink cells denote higher expression, and cyan cells denote lower expression. Row edges are color coded according to the omics data source type: green, transcriptomics; red, proteomics; and blue, metabolomics.

Coexpression analysis is employed to display expression profile similarities among entities. Figure 3 presents the visualization of a coexpression profile as a 3Omics-generated heatmap. Rows represent the input transcripts and metabolites, and the colored bars alongside each row indicate the type of input molecule. For example, blue indicates metabolites, and green indicates transcripts. The columns comprise five groups of treatments and the time points. The dendrogram on the top and left displays the similarities of rows or columns. The molecules in the lower boxed area, A, (Figure 3A) includes TARDBP, PDCD6IP, RNPEP and etc. which are mostly in the largest cluster in correlation analysis (see Figure 2, and the list of largest and second largest clusters in Additional 2: Figure S2). The majority of molecules in the upper boxed area, B, (Figure 3B) belong to the second-largest cluster in correlation analysis. In coexpression analysis, molecules connected by dendrograms have similar expression levels. However, not all nodes in the same cluster as seen in the correlation analysis are closely connected in coexpression analysis. Note that although both positive and negative correlations are likely correlated based on the correlation analysis, only positive correlations are considered in this coexpression analysis. Therefore, some molecules in the same cluster in the correlation analysis may not be located in the same box in a given coexpression analysis (heat map). For example, BCL2A1 is highly correlated with PDCD6IP in the largest cluster of the correlation analysis, but BCL2A1 is not located in the co-expression analysis in Figure 3A.

**Figure 4:**
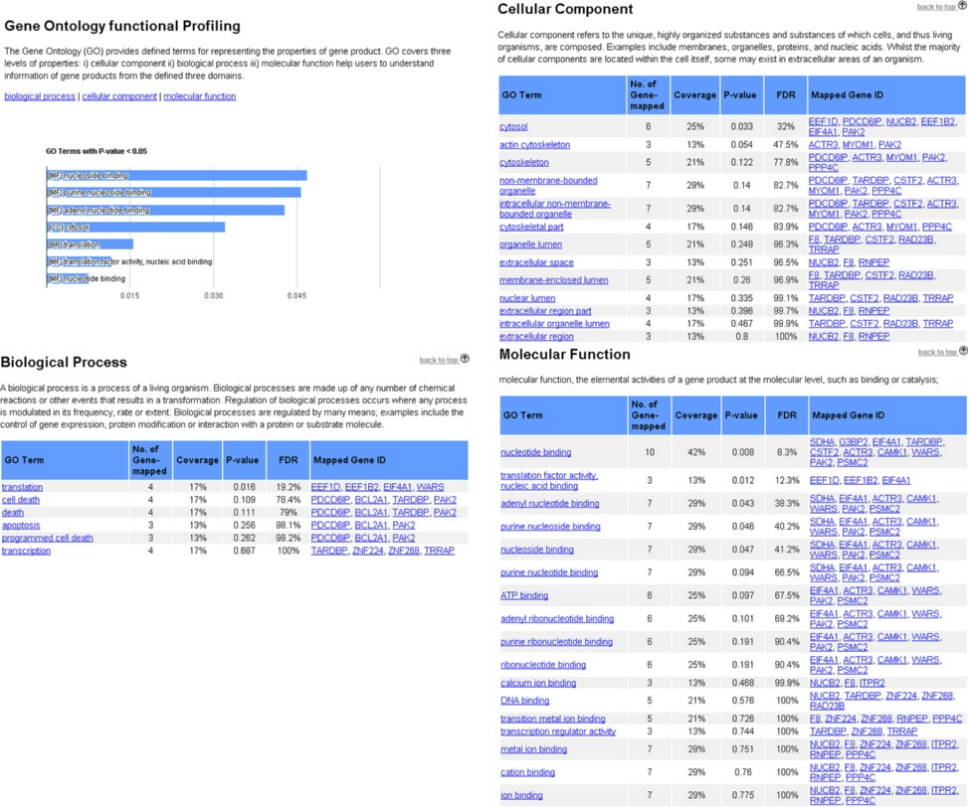
**3Omics GO Enrichment analysis.** GO terms with p-values less than 0.05 are displayed in a bar chart. Detailed results are divided into three sections corresponding to the three ontologies. Each section has a table summarizing the enriched terms with the mapped Entrez Gene IDs, the coverage of the input Gene IDs, and the p-values.

GO enrichment analysis provides defined and enriched terms to represent the properties of gene products. Based on the GO enrichment results, the functions of PDCD6IP, BCL2A1, TARDBP and PAK2 are relevant to apoptosis or cell death (Figure 4). EEF1D, EEF1B2, and EIF4A1 function as translation factors, while SDHA, G3BP2, EIF4A1, TARDBP, CSTF2, ACTR3, CAMK1, WARS, PAK2, and PSMC2 have nucleotide-binding functions. NUCB2, F8, and ITPR2 have calcium-binding abilities (Figure 4).

From the correlation network and GO-enrichment analysis, we observed that BCL2A1, PDCD6IP, and TARDBP influence apoptosis under RA and ATO treatments as well as RA and ATO combination treatment [38]. The regulation of translation factors (EEF1D, EEF1B2, and EIF4A1) may represent the regulation of tumor growth [43]. The calcium-binding abilities of many molecules reveal the calcium-dependent nature of this activity. NUCB2 is a key calcium-storage protein inside of the endoplasmic reticulum. The regulation of NUCB2 may lead to calcium-dependent reactions and/or pathways [38].

**Figure 5:**
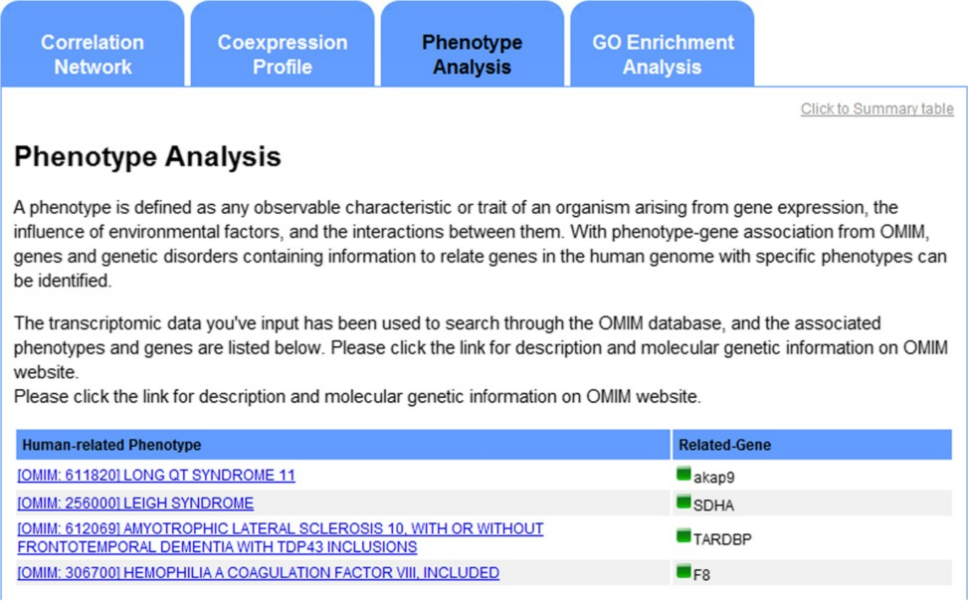
**3Omics Phenotype analysis.** Input transcriptomics data are used to query the internal 3Omics phenotype database. The matched gene-phenotype results are returned in a table. Each OMIM entry includes a hyperlink to the external OMIM database.

Phenotype analysis is performed by mapping OMIM entries with input transcripts or protein data. Each result in the returned list represents a human-related phenotype and the corresponding genes, as shown in Figure 5. The results also list the external database source and ID. The results of the phenotype analysis provide the phenotypes that are associated with the genes from the input transcriptomics data. AKAP9 is related to Long QT Syndrome. SDHA, TARDBP, and F8 are related to three other genetic diseases: Leigh Syndrome, amyotrophic lateral sclerosis 10 and hemophilia A. These genes may not be directly associated with leukemia, but the associations provide insights into possible related molecular mechanisms of these diseases.

#### Case study 2: Intra-omics analysis of urinary metabolite concentration data from 73 cancer patients measured by ^1^H NMR

To demonstrate the usefulness of KEGG and HumanCyc pathway enrichment analysis for intra-omics datasets, we used the urinary metabolome dataset from MetPA [39] to perform a KEGG/HumanCyc pathway enrichment analysis. This study investigated whether certain metabolic pathways were significantly different during muscle gain and muscle loss in cancer patients. Urine was collected from cancer patients experiencing either muscle gain or muscle loss during a three-month period, and the urine was analyzed by ^1^H NMR spectroscopy. We performed unpaired one-tailed Student’s t-tests on the urinary metabolite data to obtain p-values for each metabolite. Two datasets are required to perform a 3Omics KEGG/HumanCyc pathway enrichment analysis: one set consists of metabolites with p-values less than 0.01 (significant metabolites), and the other contains all of the metabolites included in the study (overall metabolites). These two datasets are used as the inputs for this 3Omics intra-omics analysis (intra-metabolomics) with the “Enrichment Test” (as shown in the checkbox in the lower-right corner of Figure 1).

**Figure 6:**
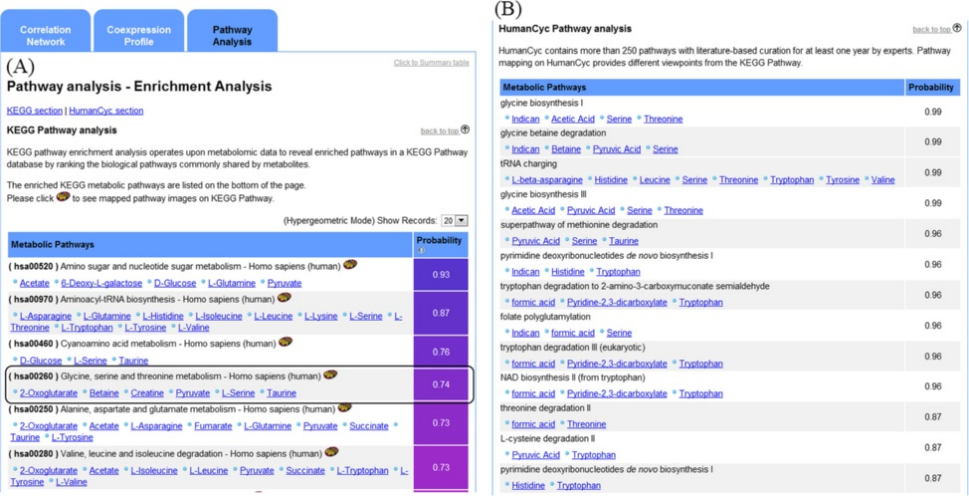
**3Omics Pathway enrichment analysis.** The enriched KEGG/HumanCyc pathways, ranked by probability from the hypergeometric test, are returned in a table. **(A)** Consistent with the original study results, the “glycine, serine and threonine metabolism pathway” (highlighted) is a top search hit, with a probability of 0.74 based on the KEGG pathway enrichment analysis. **(B)** The enriched pathways from the HumanCyc pathway enrichment are also consistent with the original study results and the KEGG Pathway enrichment and provide a significant amount of meaningful information.

KEGG/HumanCyc pathway enrichment analysis (Figure 6) returns results in tables ranked according to probability. The enriched KEGG/HumanCyc pathways, ranked by the probability computed from hypergeometric tests with the KEGG and HumanCyc pathways, are listed in the results table. The hypergeometric test is used to determine whether particular metabolic pathways significantly differ in two patient groups. Metabolites in the pathway are displayed alongside the results (Figure 6). A link to a KEGG pathway map is also generated. In this case study, the top three enriched metabolic pathways were amino sugar and nucleotide sugar metabolism, aminoacyl-tRNA biosynthesis, and cyanoamino acid metabolism. Xia et al. [39] identified the “glycine, serine and threonine metabolism” pathway as the top pathway in a pathway topological analysis; this pathway was also significant in the MetPA pathway enrichment analysis. Although the pathway enrichment analysis utilized in MetPA differs from that utilized in the KEGG pathway enrichment analysis in 3Omics, the 3Omics analysis returned a probability of 0.74 for the highly significant enriched pathways for “glycine, serine and threonine metabolism”, a finding that is consistent with the results from the original investigation. The six amino acids highlighted in the rectangular section of Figure 6A are important in the “glycine, serine and threonine metabolism” pathway.

The top enriched pathways in the HumanCyc pathway analysis as seen in Figure 6B are glycine biosynthesis I & III, glycine betaine degradation and threonine degradation II, which are also consistent with the “glycine, serine and threonine metabolism” in the KEGG pathway. It is much easier to understand how the mechanism may involve the biosynthesis of glycine or the degradation of betaine and threonine in HumanCyc pathway analysis due to the more detailed pathways categories in HumanCyc. In addition to the enrichment results, 3Omics also provides a list of metabolites with links mapped to a particular pathway for viewing, as demonstrated in the boxed area of Figure 6. An intuitive exploration of these results can be performed by clicking the hyperlink paired with each metabolite.

In addition to the 3Omics KEGG/HumanCyc enrichment analysis, users are encouraged to examine different statistical analyses. The 3Omics correlation network analysis for this case study, for example, shows that leucine and valine have higher correlations compared with other metabolites (data not shown). This observation is in accordance with the 3Omics KEGG pathway enrichment analysis, which also maps the following pathways: valine, leucine and isoleucine degradation and biosynthesis; alanine, aspartate and glutamate metabolism; amino sugar and nucleotide sugar metabolism; and cyanoamino acid metabolism.

### Limitations

Integration of multi-omics datasets in one online tool helps researchers to unravel the complete picture of a biological system. For example, Ishii et al. [44] studied the responses to environmental and genetic perturbations in *Escherichia coli* using transcriptomics, proteomics, and metabolomics data. Similarly, Trauger et al. studied the adaptation of *Pyrococcus furiosus* to a temperature shift by integrating transcriptomics, proteomics, and metabolomics analyses [45]. However, incorporating heterogeneous multi-omics datasets does not guarantee high-quality results and may yield poor results due to false-positive results.

Currently, 3Omics accepts human-only data and provides human-specific analyses, such as literature-derived information and human phenotypes from human genes/proteins. However, 3Omics works on human data with a provision for incorporating data for other species.

## Conclusions

We developed a one-click web tool for fast analysis and visualization of multi-omics data. 3Omics integrates transcriptomics, proteomics, and metabolomics datasets in combination or as single omics datasets. For integration, analysis, and visualization of multi-omics datasets, we incorporated five common features to present the information from input molecules: correlation and coexpression analysis to display the relationships among input molecules; GO-based enrichment analysis to provide information on biological events, molecular functions, and the cellular localization of transcripts and proteins; phenotypic analysis to expose related phenotypes of transcripts; and pathway-enrichment analysis to cover mapped KEGG/HumanCyc pathways in the normal mode and enriched pathways in the enrichment mode. From Case Studies 1 and 2, the results of the 3Omics analyses are consistent with the original reports based on the inter- and intra-omics datasets. Furthermore, possible mechanisms and biological functions are provided without manual editing in 3Omics. 3Omics incorporates the functionality of existing software into one piece of software, thereby simplifying data analysis and enabling users to perform a one-click integrated analysis.

## Availability and requirements

Project name: 3Omics

Project home page: http://3omics.cmdm.tw

Operating system: Platform independent

Programming language: Perl and PHP

Other requirements: Web browsers with JavaScript enabled. Microsoft Internet Explorer 7.0 or later, Google Chrome 5.0 or later, or Mozilla Firefox 2.0 or later.

License: None

Non-academic use restrictions: None

## Abbreviations

KEGG: Kyoto Encyclopedia of Genes and Genomes; OMIM: Online Mendelian Inheritance in Man; GO: Gene Ontology; iHOP: information Hyperlinked Over Proteins.

## Competing interests

The authors declare that they have no competing interests.

## Authors’ contributions

T-CK developed core functions, implemented programs, enhanced the user interface, and wrote the manuscript. T-FT developed the main format of the web interface, reviewed literature, prepared samples, and wrote the manuscript. YJT initiated the project, developed the core functions, adjusted the user interface, edited the manuscript and served as the advisor for this project. All authors read and approved the final manuscript.

## Supplementary Material

Additional file 1: Figure S1 A typical Workflow of 3Omics. After a user uploads their data to the server, the experimental data is processed by a series of analytical and visualization methods. The Name ID converter is optional for converting molecule names into database IDs. Correlation network analysis and co-expression analysis -omics analysis. Phenotypic analysis, Pathway and Gene Ontology Enrichment Analysis only utilize part of the analysis flowutilize all seven type of the analysis flow. Click here for file

Additional file 2: Figure S2 Summary Table of Clusters in the Correlation Network. All input molecules formed as clusters in correlation analysis. Each molecule has a link to external database. Transcripts are linked to NCBI Entrez Gene, proteins are linked to UniProt, and metabolites are linked to Human Metabolome. Click here for file

Additional file 3: Figure S3 Association Table of the Literature-derived Metabolites and the Proteins. Literature-derived metabolites associates with proteins are reported in this table. Each literature-derived metabolite has a link to KEGG, PubChem, HMDB, and HMO (Human Metabolome Ontology). Click here for file
