# Supplementary figures and images for: 3Omics: a web-based systems biology tool for analysis, integration and visualization of human transcriptomic, proteomic and metabolomic data
Source: BMC Syst Biol. 2013 Jul 23;7:64. doi: 10.1186/1752-0509-7-64 (PMC3723580; doi:10.1186/1752-0509-7-64)

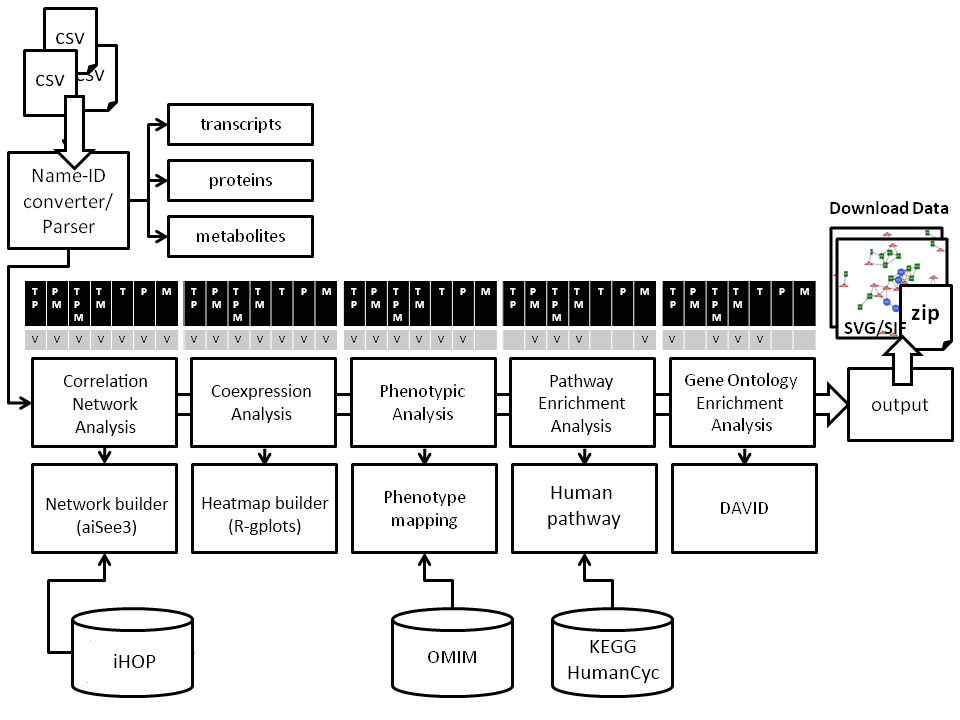

Supplement: Additional file 1: Figure S1 — A typical Workflow of 3Omics. After a user uploads their data to the server, the experimental data is processed by a series of analytical and visualization methods. The Name ID converter is optional for converting molecule names into database IDs. Correlation network analysis and co-expression analysis -omics analysis. Phenotypic analysis, Pathway and Gene Ontology Enrichment Analysis only utilize part of the analysis flowutilize all seven type of the analysis flow. [file 1752-0509-7-64-S1.png]

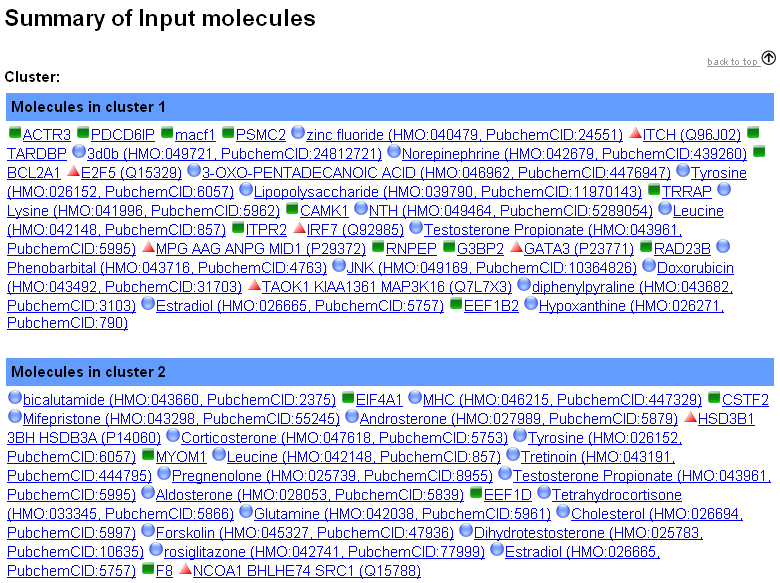

Supplement: Additional file 2: Figure S2 — Summary Table of Clusters in the Correlation Network. All input molecules formed as clusters in correlation analysis. Each molecule has a link to external database. Transcripts are linked to NCBI Entrez Gene, proteins are linked to UniProt, and metabolites are linked to Human Metabolome. [file 1752-0509-7-64-S2.png]

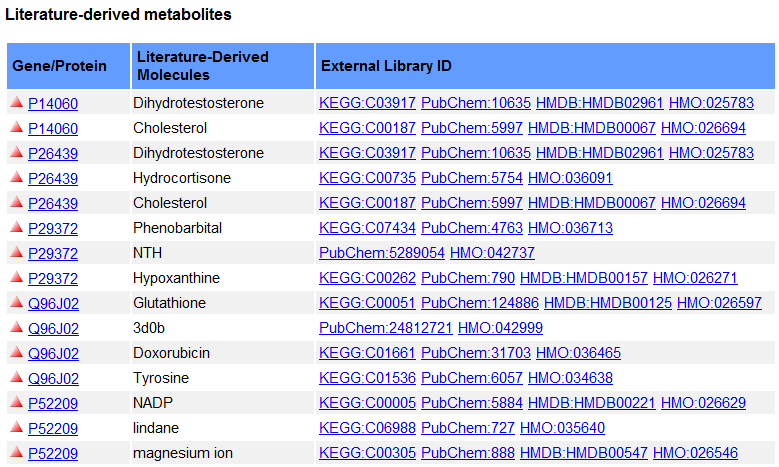

Supplement: Additional file 3: Figure S3 — Association Table of the Literature-derived Metabolites and the Proteins. Literature-derived metabolites associates with proteins are reported in this table. Each literature-derived metabolite has a link to KEGG, PubChem, HMDB, and HMO (Human Metabolome Ontology). [file 1752-0509-7-64-S3.png]
